# Applications of Non-Coding RNAs in Patients With Retinoblastoma

**DOI:** 10.3389/fgene.2022.842509

**Published:** 2022-03-31

**Authors:** Daniel Fernandez-Diaz, Cristina Rodriguez-Vidal, Paula Silva-Rodríguez, Laura Paniagua, María José Blanco-Teijeiro, María Pardo, Antonio Piñeiro, Manuel Bande

**Affiliations:** ^1^ Department of Ophthalmology, University Hospital of Santiago de Compostela, Santiago de Compostela, Spain; ^2^ Tumores Intraoculares en el Adulto, Instituto de Investigación Sanitaria de Santiago (IDIS), Santiago de Compostela, Spain; ^3^ Department of Ophthalmology, University Hospital of Cruces, Barakaldo, Spain; ^4^ Fundación Pública Galega de Medicina Xenómica, Clinical University Hospital, Santiago de Compostela, Spain; ^5^ Department of Ophthalmology, University Hospital of Coruña, A Coruña, Spain; ^6^ Grupo Obesidómica, Instituto de Investigación Sanitaria de Santiago (IDIS), Santiago de Compostela, Spain

**Keywords:** retinoblastoma, non-coding RNA, lncRNA, miRNA, circRNA, diagnostic biomarker, prognostic factor, therapeutic target

## Abstract

Retinoblastoma (RB) is the most common primary intraocular malignancy in childhood. In the carcinogenic process of neoplasms such as RB, the role of non-coding RNAs (ncRNAs) has been widely demonstrated recently. In this review, we aim to provide a clinical overview of the current knowledge regarding ncRNAs in relation to RB. Although ncRNAs are now considered as potential diagnostic biomarkers, prognostic factors, and therapeutic targets, further studies will facilitate enhanced understanding of ncRNAs in RB physiopathology and define the roles ncRNAs can play in clinical practice.

## 1 Introduction

Retinoblastoma (RB) is a retinal tumor that is the most common primary intraocular malignancy in children (Dimaras et al., 2012). RB incidence is one case per 15.000–20.000 live births, which results in approximately 9.000 new cases per year (Kivelä, 2009) without statistically significant differences according to sex or race (Rao and Honavar, 2017). Although numerous treatment techniques that allow local tumor management in RB are available, the most frightening side consequence remains the development of metastatic illness (AlAli et al., 2018). Notably, both the conservation of the eyeball and the survival of the patient with RB depend largely on the tumor stage at the time of diagnosis, and RB identification occurs substantially later in underdeveloped countries than in developed nations and results in a considerably lower survival rate and a 50%–70% fatality rate (Wessels and Hesseling, 1996; Bowman et al., 2008; Kivelä, 2009).

RB is a genetic disease in which the loss of the tumor suppressor gene *RB1* (locus 13q14.2) plays a major role (Knudson, 1971; Brantley and Harbour, 2001). RB occurs in a non-hereditary form with monocular involvement due to a somatic mutation of both *RB1* alleles and, in a hereditary form in which the ocular involvement might be single or bilateral with a first mutation of the *RB1* allele occurring in the germline and subsequently taking place the other somatic mutation (Knudson, 1971; Comings, 1973). Although inactivation of biallelic *RB1* is the main factor for the origin of RB, it is known that a small percentage of patients (approximately 2%) lack this alteration but show an abnormal amplification of the *MYCN* gene (Rushlow et al., 2013). Currently, highly precise genomic studies are expanding our understanding of this neoplasm, and it is becoming increasingly clear that additional genetic and epigenetic changes are required for RB tumorigenesis (Thériault et al., 2014). In this line, Afshar et al. found genetic alterations beyond *RB1* inactivation which correlate with more aggressive histopathological features, i.e., deleterious mutations in tumor suppressor genes (*BCOR*, *ATRX*, *ARID1A*, *FAT1*, and *MGA*) as well as amplifications in oncogenic genes (*RAF1* and *MDM4*) (Afshar et al., 2020). Similarly, Francis et al. using clinical next-generation sequencing found additional molecular alterations in all study samples (no two specimens had identical genetic pattern), being the most common non-*RB1* gene alteration *BCOR*, which was associated with poor metastases-free survival (MFS), while vitreous seeding was correlated with 16q loss and 1q gains; and in 11% of cases non-*RB1* germline mutations in other cancer-associated genes were found (Francis et al., 2021). It is also known that several chromatin regulators are misregulated in RB and play a relevant role in tumor transformation following *RB1* inactivation, including DNA methyltransferase (DNMT), Ubiquitin-like with PHD and ring finger domains 1 (UHRF1), and B lymphoma Mo-MLV insertion region 1 (BMI1) (Lee and Kim, 2021). It should be noted that, recently, several studies have shown relevant findings in this field, on the one hand, they were able to develop in the laboratory induced pluripotent stem cells (iPSCs) from germline *RB1* mutated patients and optimize a retinal organoid culture method to obtain human RB; on the other hand, they established the necessary tools to induce, in human embryonic stem cells (hESCs), *RB1* mutations to obtain RB indistinguishable from those of patient-derived iPSCs. Moreover, the histopathological, cytogenetic, molecular, epigenetic and clonal aspects of organoid-derived RB were consistent and similar to patients’ RB, so that can be used to study etiopathogenesis as well as new therapeutic approaches (Liu et al., 2020, 2021; Norrie et al., 2021).

Epigenetics is widely recognized to play a fundamental role in ocular pathologies, a role that encompasses, among several other mechanisms, regulation through non-coding RNAs (ncRNAs) (Lee, 2012; Schmitz et al., 2016). NcRNAs constitute the vast majority of the human genome, and despite lacking the capacity for translation into proteins, ncRNAs more potently influence numerous biological functions than do coding RNAs (Guzel et al., 2020).

Distinct classes of ncRNAs have been identified, including long non-coding RNAs (lncRNAs), microRNAs (miRNAs), and circular RNAs (circRNAs); these ncRNAs contribute to the appearance, pathogenesis, and evolution of various eye diseases, such as cataracts, premature retinopathy, age-related macular degeneration, eye tumors, etc. (Yan et al., 2014; Li et al., 2016; Wawrzyniak et al., 2018; Guo et al., 2019; Zhang et al., 2019).

**FIGURE 1:**
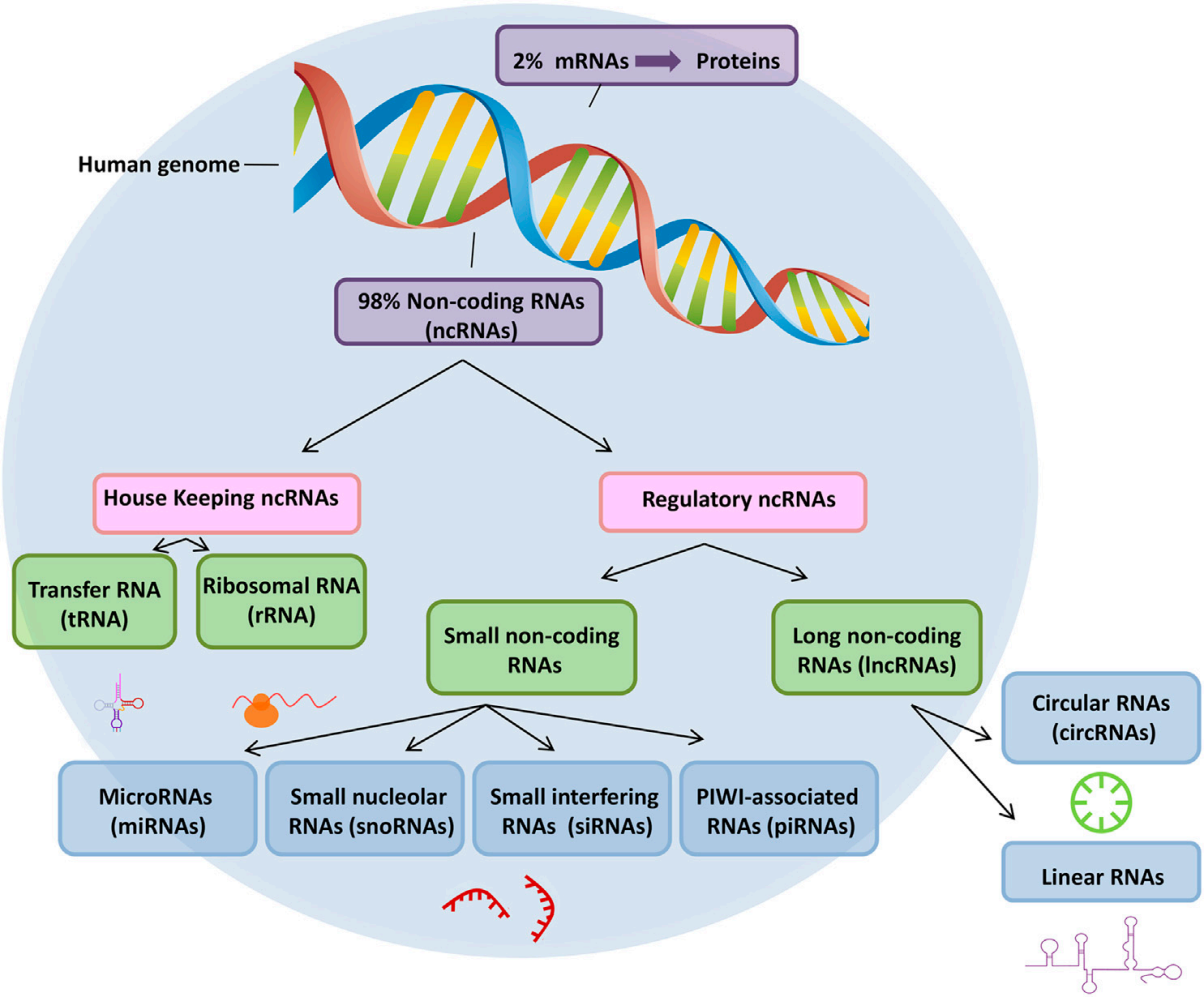
ncRNA classifications. RNAs are classified into two broad categories: messenger RNA (mRNA) and non-coding RNA (ncRNA).

In recent times, an increasing number of studies are focusing on the role of ncRNAs in RB. In this line, ncRNAs are considered to be relevant and novel biomarkers not only for early diagnosis of this neoplasm, but also for offering a therapeutic and prognostic approach for enhancing the quality of life and survival of patients with RB (Figure 1).

## 2 LncRNAs and CircRNAs in RB

LncRNAs, which are ncRNAs composed of >200 nucleotides, are crucial in neoplastic processes (Chu et al., 2011; Gibb et al., 2011; Prensner and Chinnaiyan, 2011), including RB development (Li et al., 2016; Yang and Wei, 2019; Zhang et al., 2019).

To date, diverse molecules have been discovered in the context of lncRNAs exhibiting oncogenic activity. Hao et al. (Hao et al., 2018) observed that actin filament-associated protein 1-antisense RNA 1 (AFAP1-AS1) was overexpressed and associated with tumor size, choroidal infiltration, and optic nerve invasion as an independent unfavorable prognostic factor (*p* = 0.012), and this resulted in a marked decrease in the overall survival (OS) of the RB patients (*p* < 0.001). Similar findings were reported by Su et al. (Su et al., 2015) in the case of BRAF-activated non-coding RNA (BANCR); although BANCR plays a tumor-suppressing role in other types of cancers, it was found to be overexpressed in RB and to affect prognosis, with a median of 20 months of OS recorded for patients with high expression of BANCR as compared to a median of >60 months for patients with low expression (*p* < 0.001). Moreover, antisense non-coding RNA in the INK4 locus (ANRIL), which was shown to be upregulated in RB samples, favored proliferation, migration, and cell invasion and reduced apoptosis by producing, among other effects, inhibition of the *ATM-E2F1* pathway (Yang and Peng, 2018). Wang et al. (Wang et al., 2018) demonstrated that differentiation-antagonizing non-protein-coding RNA (DANCR) was overexpressed in RB cell lines and tissues, and that by stimulating proliferation, migration, invasion, and epithelial-mesenchymal transition (EMT) by acting as a competing endogenous RNA (ceRNA) of the miRNAs miR-34c and miR-613, DANCR worsened disease-free survival (DFS) (*p* = 0.0084) and OS (*p* = 0.0056). The lncRNA colon cancer-associated transcript 1 (CCAT1) induced similar changes in RB samples, in this case through the miR-218-5p/*MTF2* axis (Zhang et al., 2017; Meng et al., 2021). Accordingly, tumoral overexpression of HOX antisense intergenic RNA (HOTAIR) was shown to promote tumorigenesis and decrease OS (*p* = 0.002) through the miR-613*/c-Met* axis and the *Notch1* pathway and act as an independent prognostic factor (*p* < 0.05) (Dong et al., 2016; Yang et al., 2018). Furthermore, increased levels of promoter of CDKN1A antisense DNA damage-activated RNA (PANDAR), a newly identified lncRNA, were reported to be associated with unfavorable clinicopathological characteristics such as optic nerve invasion and advanced International Intraocular Retinoblastoma Classification (IIRC) stage; this was partly due to PANDAR inhibiting apoptosis when interacting with the *Bcl-2/caspase-3* pathway (Sheng et al., 2018). Similarly, evidence has been reported for the clinical relevance of testis-associated highly conserved oncogenic long non-coding RNA (THOR), whose overexpression enhances the malignant phenotype of RB in association with the oncogene *c-Myc* and the gene encoding *insulin-like growth factor 2 mRNA-binding protein 1* (*IGF2BP1*) (Shang, 2018). Lastly, small nucleolar RNA host gene 14 (SNHG14) and plasmacytoma variant translocation 1 (PVT1), two other lncRNAs that promote tumor aggressiveness and lower OS (*p* < 0.001 and *p* < 0.032), respectively, were reported to act by sponging, respectively, the miRNAs miR-124 and miR-488-3p (Wu et al., 2019; Sun et al., 2020).

As compared to lncRNAs showing oncogenic activity, the lncRNAs that play a tumor-suppressing role in RB have been investigated to a lesser extent; however, recent studies have started to elucidate this function of lncRNAs and have made notable contributions. Shang et al. (Shang et al., 2018) demonstrated that BDNF antisense RNA (BDNF-AS) was downregulated in RB samples and correlated with lower OS (*p* = 0.009), thus emerging as a statistically significant independent factor for the prognosis of these patients (*p* < 0.005). Similarly, lncRNA maternally expressed gene 3 (MEG3) showed decreased expression levels in RB tissues and cell lines, and this was negatively associated with metastatic disease development and the IIRC stage due to MEG3 regulation of the *Wnt/β-catenin* and *p53* pathways. The diminution in MEG3 levels also showed a statistically significant association with the decrease in patient survival, as revealed through Kaplan-Meier analysis (*p* < 0.001) (Gao and Lu, 2016, 2017). Moreover, metallothionein 1J pseudogene (MT1JP), despite playing an oncogenic role in tumors such as hepatoblastoma, colon, breast, prostate, and non-small cell lung cancers, functions as a tumor suppressor in RB. Bi et al. found that MT1JP was underexpressed and negatively modulated the activity of the *Wnt/β-catenin* axis, which correlated with the invasion of the optic nerve, the IIRC stage, and the appearance of metastasis in RB cases (Bi et al., 2018).

Although lncRNAs are known to exhibit divergent behaviors in distinct types of tumors, the role of the lncRNA H19 in RB remains debated. Zhang et al. demonstrated that H19 expression in RB was decreased and that the lncRNA acted as a tumor suppressor at the levels of proliferation, cell cycle, and apoptosis by interacting with the miR-17-92 cluster (Zhang et al., 2018). By contrast, Qi et al. and Li et al. found that H19 was overexpressed in tumor samples and played an oncogenic role by promoting cell proliferation, migration, and invasion and reducing apoptosis, which ultimately led to increased tumor size, invasion of the optic nerve, and choroidal infiltration; this resulted in a shorter OS (*p* = 0.006), and the H19 expression level emerged an independent prognostic factor (*p* < 0.001) (Li et al., 2018; Qi et al., 2019).

Supplementary Table S1 lists all notable unregulated lncRNAs in detail, including their chromosomal location, molecular targets, signaling pathways in which they intervene, type of study sample, mode of action, influence on OS, degree of prognostic factor, role in other types of cancer, and the technical validation methods used for their characterization.

CircRNAs represent a newly identified molecular group of non-coding endogenous RNAs that feature a loop shape and are less susceptible to degradation than other RNAs (Jeck and Sharpless, 2014; Vicens and Westhof, 2014). The functions of circRNAs include acting as miRNA sponges, eliminating the initiation codons of mature mRNAs to limit protein translation, controlling alternative splicing, and interacting with RNA-binding proteins (Salzman et al., 2012; Zhang et al., 2013; Salzman, 2016). Current evidence indicates that circRNAs play key roles in the cellular processes of several ocular diseases, including RB (Wawrzyniak et al., 2018; Guo et al., 2019). Accordingly, an overall decrease in circRNA expression levels was demonstrated in RB by Lyu et al. (Lyu et al., 2019a), who further reported that TET1-hsa_circ_0093996 expression was downregulated in both primary and recurrent RB samples, which led to an increase in miR-183 levels and ultimately to a reduction in the expression of the tumor suppressor programmed cell death 4 (*PDCD4*). Xing et al. (Xing et al., 2018) demonstrated that hsa_circ_0001649 was also underexpressed in RB, and that this favored tumorigenesis by regulating cell proliferation and apoptosis through the *Akt/mTOR* signaling pathway; moreover, the circRNA downregulation was found to be correlated with increased tumor size, more advanced clinical stage, and shorter OS (*p* = 0.005), demonstrating the potential usefulness of this circRNA as a prognostic biomarker and therapeutic target in RB. Recent studies (Zhang et al., 2021; Zheng et al., 2021) have shown highly expression in RB cell lines and tissues of circ-FAM158A and circ_0075804, which promote malignant behavior through the miR-138-5p/*SLC7A5* and miR-138-5p/*PEG10* axis, respectively.

In short, numerous investigations have demonstrated the contribution of both lncRNAs and circRNAs in the pathogenesis of RB. It is true that, to date, the number of known lncRNAs is much larger and the field of study of circRNAs is more limited. Therefore, it is necessary to continue researching and expanding our knowledge of these molecules in RB.

## 3 MiRNAs in RB

### 3.1 Dysregulated Expression of MiRNAs

Aberrant expression of miRNAs, which represent a type of small ncRNAs that are 17–22 nucleotides long, critically affects the pathophysiology and development of RB (Singh et al., 2016; Delsin et al., 2019). Among ncRNAs, miRNAs have been investigated most comprehensively, and novel compounds targeting miRNAs are being developed continuously and rapidly. Supplementary Tables S2, S3 summarize the wide variety of miRNAs discovered to date; the miRNAs are classified into two categories (according to tumor-suppressing and oncogenic actions), and the tables also list the miRNA molecular targets, axes of action, and cellular functions, as well as the study sample type, the effect of the miRNAs on the OS of patients, and the technical validation methods used.

**FIGURE 2:**
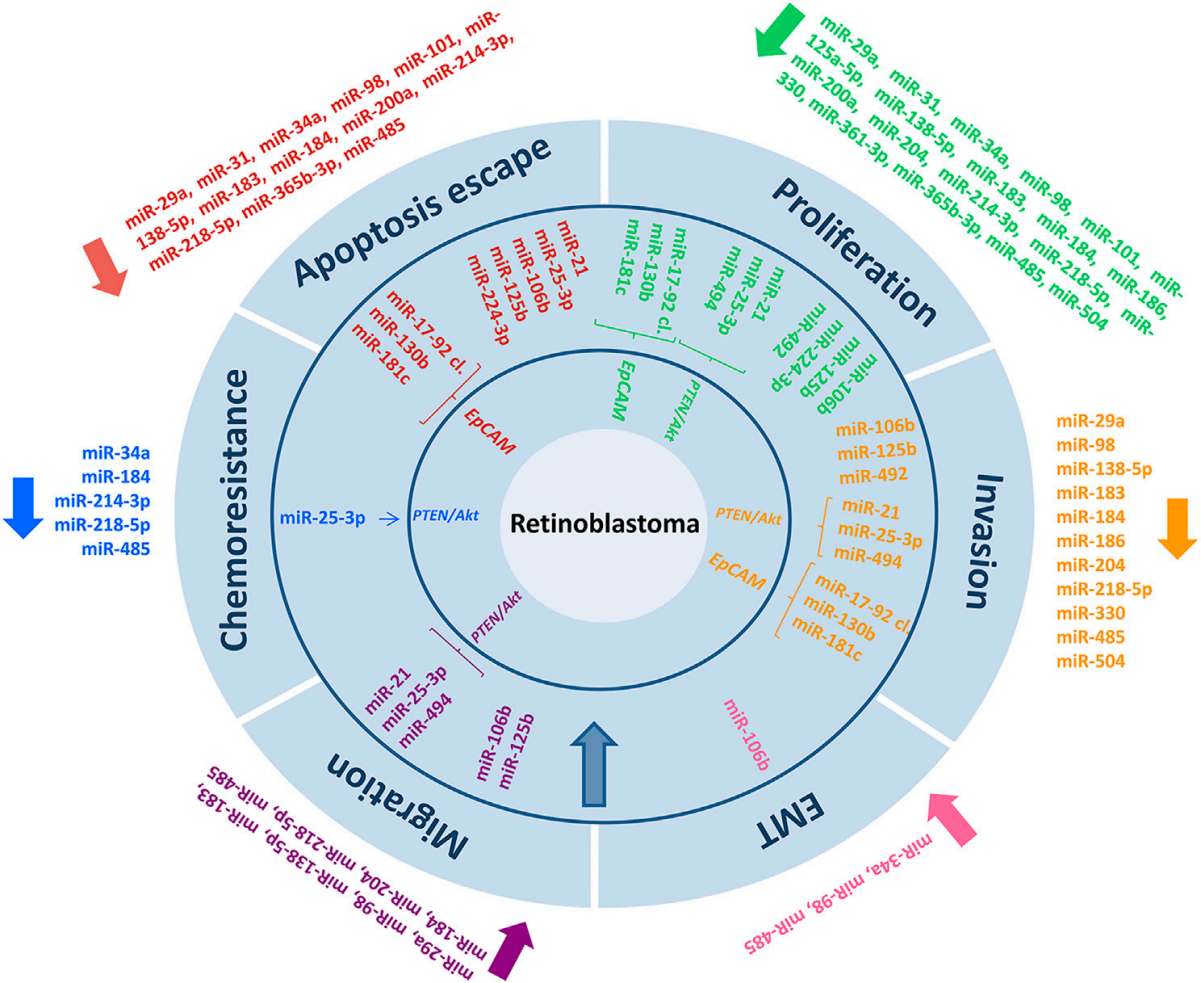
In retinoblastoma cancer, microRNA biomarkers and their related targeting mechanisms have been identified.

Among miRNAs identified as tumor suppressors, Guo et al. (Guo et al., 2019) and Lei et al. (Lei et al., 2014) demonstrated that miR-98 and miR-101 were underregulated in RB samples, and this was correlated with unfavorable clinical-histopathological characteristics (tumor size, choroidal/orbital infiltration, and optic nerve invasion) and negatively affected prognosis. Although similar repercussions were recorded with both molecules, the miRNAs act through distinct pathways: whereas miR-98 influences *insulin-like growth factor-1 receptor* (*IGF1R*), miR-101 inhibits the expression of the oncogenic *enhancer of zeste homolog 2* (*EZH2*) protein. Zhao and Cui (Zhao and Cui, 2019) demonstrated that miR-361-3p was markedly downregulated in RB tissues, RB serum, and RB cell lines as compared with the levels in normal retinal tissues and normal serum, and that the miRNA functions in suppressing tumorigenesis and progression by acting on the *sonic hedgehog* (*SHH*) signaling pathway; this is the opposite of what occurs in cervical cancer, where miR-361-3p has been shown to serve as an oncogene in cancer progression by enhancing cell proliferation and invasion. Another miRNA that potently affects the prognosis of patients with RB is miR-485 (Lyu et al., 2019b); miR-485 was underexpressed in patient samples and RB cell lines and this downregulation influenced not only cell proliferation, migration, and invasion, but also apoptosis, chemoresistance, and EMT through *Wnt3*, which were all increased when the miRNA was downregulated. Intriguingly, both miR-31 and miR-200a were reported to be underexpressed in one study, and an increase of their expression levels failed to limit the expansion of a relatively less aggressive RB cell line (Weri-Rb1) but restricted the growth of the highly proliferative Y79 cell line (Montoya et al., 2015). Several other miRNAs have also been shown to be expressed at low levels in RB. For example, Martin et al. (Martin et al., 2013) confirmed the deregulation of various miRNAs, including miR-22, miR-129-3p, miR-129-5p, miR-382, and miR-504.

In terms of miRNAs that produce a tumorigenic effect, in 2009, Zhao et al. (Zhao et al., 2009) identified a range of miRNAs showing elevated expression levels in RB, including miR-129-1, miR-129-2, miR-198, miR-373, miR-492, miR-494, miR-498, miR-503, miR-513-1, miR-513-2, and miR-518c. The miR-17-92 cluster, which lies near the minimal common region of gain in RB, is one of the best-known miRNA clusters (Kandalam et al., 2012; Jo et al., 2014); the miRNA cluster promotes cell proliferation and invasion while suppressing apoptosis, and its action is modulated through interactions with *epithelial cell adhesion molecule* (*EpCAM*) and *signal transducer and activator of transcription 3* (*STAT3*). Intriguingly, *EpCAM* interacts with not only the miR-17-92 cluster, but, as shown by Beta et al. (Beta et al., 2014), also with several oncogenic miRNAs, such as miR-130b and miR-181c. In 2019, Wan et al. (Wan et al., 2019) reported that miR-25-3p, which plays an oncogenic role in other cancers such as renal tumors, triple-negative breast cancer, melanoma, and non-small cell lung cancer, also holds negative implications in the case of RB; miR-25-3p promotes tumor malignancy by enhancing proliferation, migration, EMT, *in vitro* cell invasion, and *in vivo* tumor formation, all of which increase *Akt* phosphorylation by suppressing *phosphatase and tensin homolog* (*PTEN*). Moreover, miR-25-3p increases resistance to chemotherapeutic drugs Figure 2.

### 3.2 MiRNAs as Potential Biomarkers for RB

As mentioned in Section 1, a delay in diagnosis negatively influences the ocular and vital prognosis of patients with RB. Circulating miRNAs serve as mediators of intercellular communication and are either transported by ribonucleoproteins or encapsulated in exosomes for protection against degradation (Hunter et al., 2008; Bail et al., 2010). Therefore, liquid biopsy has emerged as an accessible, non-invasive tool that provides considerable information regarding tumor diagnosis, therapeutic response, and prognosis (Mitchell et al., 2008; Heitzer et al., 2015; Delsin et al., 2019; Lande et al., 2020). Consequently, the early detection of biomarkers is considered a crucial milestone, and in this context, the study of miRNAs in body fluids, such as plasma or serum, is of substantial interest.

Beta et al. (Beta et al., 2013) examined 14 serum samples from healthy people in relation to a comparable number of serum samples from patients with RB, and the results showed that 24 miRNAs were underexpressed and 21 were overexpressed in the patients group; Beta et al. also associated the serum miRNA profiles with those of published tumor samples, which led to the identification of 33 miRNAs (8 downregulated and 25 upregulated), although the overexpression of only miR-17, miR-18a, and miR-20a could be verified. In a later study, Liu et al. (Liu S.-S. et al., 2014) examined 65 plasma samples from patients with RB and healthy people, and the results confirmed the down-regulation of miR-21, miR-320, and let-7e, as well as the association of the miRNA levels with tumor growth. In a more recent study (Castro-Magdonel et al., 2020), the circulating miRNome was analyzed in 12 children with RB and 12 healthy controls of the same age, which resulted in the detection of a plasma signature of 19 miRNAs (miR-378h, miR-455-3p, miR-1281, miR-3201, miR-3613-3p, miR-3921, miR-4507, miR-4508, miR-4529-3p, miR-4668-5p, miR-4707-5p, miR-4750-3p, miR-4763-3p, miR-6069, miR-6085, miR-6511b-5p, miR-6777-5p, miR-6794-5p, and miR-8084) in all the patients with a discriminatory capacity against the controls; moreover, 14 of the miRNAs were also detected in the corresponding primary tumors. In the same study, 4 miRNAs associated with male sex (miR-378h, miR-4706, miR-4763-3p, and miR-6511b-5p) and 5 miRNAs associated with female sex (miR-1469, miR-3620-5p, miR-6088, miR-6089, and miR-6794-5p) were discovered, which suggested the existence of a distinct hormonal functional axis. Pathway analysis of these miRNAs revealed hormonal functions, including functions related to estrogen, thyroid signaling pathways, and testosterone biosynthesis. Similarly, reduced expression levels of miR-144 were demonstrated in both tissue and serum samples from patients with RB, and these levels were positively correlated with each other and associated with tumor size, metastasis development, and lower OS (*p* = 0.0065) and DFS (*p* = 0.0331) (Zheng et al., 2020).

In recent times, anterior chamber paracentesis to obtain aqueous humor (AH) is becoming a study technique of great interest due to the information it provides. In the same way that allows the analysis of miRNAs in non-oncologic ophthalmologic diseases (Grieco et al., 2020; Zhu et al., 2020; Kosior-Jarecka et al., 2021), it is also considered a great alternative in tumor biopsy for RB (Gudiseva et al., 2019). Numerous publications have studied in AH the presence of RB biomarkers such as Lactate Dehydrogenase (LDH), Neuron-Specific Enolase (NSE) and Survivin among others (Ghiam et al., 2019). From the genetic point of view, Berry et al. demonstrated in several studies (Berry et al., 2017, 2018) the presence of detectable levels of nucleic acids, including cell-free DNA (cfDNA), RNA and miRNA, being these first ones representative of the genomic status of tumor samples; as well as the fact that the presence of 6p gain and *MYCN* amplification are indicators of worse prognosis. Furthermore, as Gerrish et al. (Gerrish et al., 2019), the possibility of detecting the specific *RB1* mutation in AH was found. Recent publications (Berry et al., 2020; Xu et al., 2021) have prospectively validated these results and even shown that chromosomal changes are more frequently detected in AH than in blood, concluding that AH is superior as a liquid biopsy in clinical setting. Advances in biopsy of AH, as well as future characterization of ncRNAs in it, will allow for better diagnostic and prognostic management applied to patients with RB.

## 4 Therapeutic Potential of NcRNAs in RB

The manipulation of ncRNAs and their regulators is considered a novel approach for RB treatment (Mirakholi et al., 2013; Golabchi et al., 2018). Examples of the molecules used for this manipulation include agents that stimulate the production of tumor suppressor ncRNAs or compounds that inhibit the expression of ncRNAs with oncogenic activities, such as antagonists targeting miR-130b and miR-181c (Beta et al., 2014). Regulation of ncRNAs involved in the physiology of chemoresistance would enable the development of highly personalized tumor therapy, thus avoiding the use of ineffective drugs from the time of therapy initiation. Previous studies showed that underexpression of miR-15a, miR-16, miR-34a, miR-3163, and the let-7 family and upregulation of miR-18a, miR-19b, miR-106a, and miR-198 are associated with chemotherapy resistance (Mitra et al., 2012; Liu K. et al., 2014; Jia et al., 2016). Moreover, other studies found that overexpression of miR-184 and miR-214-3p increased tumor chemosensitivity by triggering apoptosis, and that silencing of urothelial carcinoma-associated onco-lncRNA 1 (UCA1) also increased the susceptibility of cells to chemotherapy (He et al., 2019; Yang et al., 2020a; Yang et al., 2020b).

Hypoxia is a crucial component of the tumor microenvironment and is related to chemoresistance. *Hypoxia-inducible factor* (*HIF*) has been shown to influence a group of miRNAs known as hypoxia-regulated miRNAs, including miR-30c-2, miR-125a-3p, miR-181b, miR-491-3p, and miR-497 (Xu et al., 2011). Under hypoxic conditions, miR-320 was found to be capable of controlling autophagy in RB cells through *HIF-1*. Because of its importance, hypoxia is considered a therapeutic target in advanced RB cases (Liang et al., 2017). Another area of research is the development of targeted therapies against miRNAs in cancer stem cells, which represent a subpopulation of tumor cells that show increased resistance to cytotoxins and can lead to tumor recurrence (Mirakholi et al., 2013).

Among the new therapeutic possibilities in RB also are nucleic acid-based therapies, in which ncRNAs can function as targets for drug development (Chai et al., 2021). In this context, He et al., in 2020 demonstrated in a promising study that GapmeR-mediated lncRNA retinoblastoma associated transcript-1 (RBAT1) silencing inhibited tumorigenesis in RB, and given that GapmeR antisense oligonucleotides can freely cross the blood-eye barrier, antisense drugs targeting this lncRNA may be an interesting therapeutic option for RB patients (He et al., 2020). In a similar vein, Chai et al. demonstrated that targeted silencing of lncRNA GALNT8 antisense upstream 1 (GAU1) *via* the histidine-lysine-rich polymer (HKP)-encapsulated intraocular method showed therapeutic efficacy in RB (Chai et al., 2018). However, to date, results have generally come only from preclinical studies; therefore, the development of clinical trials based on this treatment modality is needed (Chai et al., 2021; Lee and Kim, 2021).

Because poor systemic delivery limits the use of ncRNAs as therapeutic agents, novel molecular encapsulation mechanisms and alternative administration routes are being investigated. Thus, research is being conducted on direct intravitreal injection of ncRNA in the same manner as the studies being conducted on injecting chemotherapeutic drugs into the vitreous cavity (Bai et al., 2011; Ghassemi et al., 2014; Chung et al., 2016). The direct effect of the ncRNAs on tumor cells and the low endonuclease activity found in vitreous humor are two benefits of this treatment technique (de Fougerolles et al., 2007).

In summary, although promising results have been obtained in these studies on ncRNAs, clinical application of the results will be challenging because of the complexity of the mechanisms surrounding ncRNAs, including the lack of target specificity, as well as other factors, such as the finding that a drug of interest selectively reaches the axis or route of action of an ncRNA at the level of the tumor tissue (Adams et al., 2017).

## 5 Conclusion/Discussion

Our aim in this review was to provide a comprehensive clinical overview of the current knowledge regarding ncRNAs in relation to RB, the most common primary intraocular malignancy in childhood. It is now well known that the deregulation of different types of ncRNAs is linked to a wide variety of pathophysiological processes such as proliferation, migration, invasion, apoptosis and even therapeutic response. Thus, tumour cell viability and, ultimately, patient survival will be greatly influenced by ncRNAs. Even so, it should be remembered that the functioning of these molecules sometimes seems uncertain, and can be diametrically opposed depending on the type of neoplasm, and even their role within the same tumour disease could be controversial. This fact highlights the need to continue characterizing the different classes of ncRNAs, as well as their pathways of action.

Future studies will undoubtedly further elucidate the role of each of the ncRNA molecules and the entire epigenetic sphere that surrounds them. The integration of the findings of these studies and their respective approaches will contribute to a better understanding and clinical management of patients with RB.
