# Supplementary material for: Applications of Non-Coding RNAs in Patients With Retinoblastoma
Source: Front Genet. 2022 Mar 31;13:842509. doi: 10.3389/fgene.2022.842509 (PMC9008704; doi:10.3389/fgene.2022.842509)
Supplement: Supplementary file 1 [file Table1.docx]

Table 1S: lncRNAs involved in RB pathogenesis.

| **lncRNA** | **Location^†^** | **Expression** | **Target Mechanism** | **Sample Type** | **Cellular Events** | **Survival** | **Prognostic Factor** | **Role** **in Other Cancers** | **Validation Method** | **Reference** |
| --- | --- | --- | --- | --- | --- | --- | --- | --- | --- | --- |
| AFAP1-AS1 | 4p16.1 | Overexpressed | - | RB cell lines (Weri-Rb1, Y79) and tumor tissues | Promotes proliferation, migration, and invasion | Shorter  OS  *p* < 0.001 | Independent  prognostic factor  *p* = 0.012 | Oncogenic role: lung cancer, hepatocellular carcinoma, cholangiocarcinoma, colorectal cancer, gastric cancer, pancreatic cancer, oesophageal squamous cell carcinoma, renal cell carcinoma, gallbladder cancer, ovarian cancer, tongue squamous cell carcinoma, nasopharyngeal carcinoma, and thyroid  cancer | RT-PCR, PA, MA, IA | (Hao et al., 2018) |
| ANRIL | 9p21.3 | Overexpressed | *ATM-E2F1, miR-99a/c-Myc, miR-99a/JAK/STAT* and *miR-99a/PI3K/AKT* pathways | RB cell lines (HXO-RB44, Y79) and tumor tissues | Promotes cell viability, migration, and invasion | - | - | Oncogenic role: pancreatic cancer,  osteosarcoma,  bladder cancer, and prostate cancer | RT-PCR, PA, MA, IA, AA, WB, LUCA, IF | (Yang and Peng, 2018; Wang et al., 2019b) |
| BANCR | 9q21.12 | Overexpressed | *MAPK* and *NF-kB* pathways | RB cell lines (Weri-Rb1, Y79) and tumor tissues | Promotes proliferation, migration, and invasion | Shorter  OS  *p* < 0.001 | Independent  prognostic factor  *p* < 0.040 | Oncogenic role: melanoma, papillary  thyroid carcinoma, and colorectal cancer  Tumor suppressive role: small and non-small cell lung cancer | RT-PCR, PA, MA, IA | (Su et al., 2015) |
| BDNF-AS | 11p14.1 | Underexpressed | *CDC42*, *cyclin E* and *BDNF* | RB cell lines (Weri-Rb1, Y79, SO-RB50, HXO-RB44, Rb116, Rb143) and tumor tissues | Inhibits proliferation, and migration | Shorter  OS  *p* = 0.009 | Independent  prognostic factor  *p* < 0.05 | Oncogenic role: lung cancer, and ovarian cancer | RT-PCR, PA, MA, WB | (Shang et al., 2018) |
| CCAT1 | 8q24.21 | Overexpressed | *miR-218-5p/MTF2* axis | RB cell lines (Weri-Rb1, Y79, SO-RB50) and tumor tissues | Promotes proliferation, migration, invasion, and EMT  Inhibits cell apoptosis | - | - | Oncogenic role: colon cancer, liver cancer, gallbladder cancer, and gastric cancer | RT-PCR, PA, MA, IA, AA, WB, LUCA | (Zhang et al., 2017; Meng et al., 2021) |
| CYTOR  (LINC00152) | 2p11.2 | Overexpressed | *Caspase-3, caspase-8, ki-67, Bcl-2* and *MMP-9* | RB cell lines (SO-RB50, Y79) and tumor tissues | Promotes proliferation, colony formation, migration, and invasion  Inhibits cell apoptosis | - | - | Oncogenic role: gastric cancer, colorectal cancer, breast cancer, lung adenocarcinoma, glioma, clear cell renal cell carcinoma, tongue squamous cell carcinoma, gallbladder cancer, and hepatitis B virus-associated hepatocellular carcinoma | RT-PCR, PA, MA, IA, AA, WB, ATM | (Li et al., 2018b) |
| DANCR | 4q12 | Overexpressed | *Notch* pathway, *miR-34c* and *miR-613/MMP-9* axis | RB cell lines (Weri-Rb1, Y79, SO-RB50, HXO-RB44) and tumor tissues | Promotes proliferation, migration, invasion, and EMT | Shorter  OS  *p* = 0.056 | - | Oncogenic role: colorectal cancer | RT-PCR, PA, MA, IA, WB, LUCA, ATM | (Wang et al., 2018a) |
| FAM238B (LINC00202) | 10p12.1 | Overexpressed | *miR-3619-5p/RIN1*axis | RB cell lines (Weri-Rb1, Y79, SO-RB50, HXO-RB44) and tumor tissues | Promotes proliferation, migration, and invasion | Shorter  OS  *p* = 0.0227 | - | Oncogenic role: renal cancer | RT-PCR, PA, MA, IA, WB, LUCA | (Yan et al., 2019) |
| FEZF1-AS1 | 7q31.32 | Overexpressed | *miR-1236-3p* and *miR-363-3p/PAX6* axis | RB cell lines (Weri-Rb1, Y79, SO-RB50, RBL-13) and tumor tissues | Promotes cell viability, migration, invasion, and EMT  Inhibits cell apoptosis | Shorter  DFS  *p* < 0.001 | Independent prognostic  factor  *p* = 0.001 | Oncogenic role: hepatocellular carcinoma, gastric cancer, lung adenocarcinoma, and breast cancer | RT-PCR, PA, MA, IA, AA, WB, LUCA, IHC, ATM | (Quan and Wang, 2019; Zhang et al., 2020; Liu et al., 2021) |
| H19 | 11p15.5 | Overexpressed    Underexpressed | *miR-143/RUNX2* axis and *PI3K/Akt/mTOR* pathways  *miR-17-92 cluster*, *p21* and *STAT3* pathways | RB cell lines (Weri-Rb1, Y79) and tumor tissues  RB cell lines (Weri-Rb1, Y79, SO-RB50) and tumor tissues | Regulates proliferation, migration, and invasion | Shorter  OS  *p* = 0.006  - | Independent prognostic  factor  *p* < 0.001  - | Oncogenic role: gastric cancer, colorectal cancer, glioma, and bladder cancer  Tumor suppressive role: Wilms tumor  . | RT-PCR, PA, MA, IA, AA, WB, LUCA, ATM  RT-PCR, PA, AA, WB, IF | (Li et al., 2018a; Qi et al., 2019)  (Zhang et al., 2018) |
| HOTAIR | 12q13.13 | Overexpressed | *Notch1* pathway and *miR-613/c-Met* axis | RB cell lines (HXO-RB44, Y79, SO-RB50, Weri-Rb1) and tumor tissues | Promotes cell viability, proliferation, invasion, and EMT  Inhibits cell apoptosis | Shorter  OS  *p* = 0.002 | Independent  prognostic factor  *p* < 0.05 | Oncogenic role: breast cancer, liver cancer, colorectal cancer,  oesophageal squamous cancer, pancreatic cancer, non‐small cell lung  cancer, and cervical cancer | RT-PCR, PA, IA, AA, WB, LUCA, ATM | (Dong et al., 2016; Yang et al., 2018) |
| HOXA11-AS | 7p15.2 | Overexpressed | *miR-506-3p/NEK3* axis | RB cell lines (HXO-RB44, Y79, SO-RB50, Weri-Rb1) and tumor tissues | Promotes proliferation  Inhibits  cell apoptosis | - | - | Oncogenic role: renal cancer, hepatocellular carcinoma,  laryngeal squamous cell carcinoma, non-small cell lung cancer, glioma, breast cancer, osteosarcoma, and  gastric cancer  Tumor suppressive role: ovarian cancer, and colorectal cancer | RT-PCR, PA, AA, LUCA | (Han et al., 2019) |
| MALAT1 | 11q13.1 | Overexpressed | *miR-124/STX17*, *miR-20b-5p/STAT3*, *miR-655-3p/ATAD2* and *miR-124/Slug* axis | RB cell lines (HXO-RB44, Y79, SO-RB50, Weri-Rb1) and tumor tissues | Promotes proliferation, migration, invasion, and EMT  Inhibits cell apoptosis | - | - | Oncogenic role: pancreatic ductal adenocarcinoma, hepatocellular carcinoma, lung cancer, renal cancer, bladder cancer, and glioma | RT-PCR, PA, MA, IA, AA, WB, LUCA, IHC, ATM | (Huang et al., 2018; Liu et al., 2018; Wang et al., 2020b; Zhao et al., 2021) |
| MEG3 | 14q32.2 | Underexpressed | *Wnt/β-catenin* and  *p53* pathways | RB cell lines (HXO-RB44, Y79, SO-RB50, Weri-Rb1) and tumor tissues | Suppresses proliferation  Promotes  apoptosis | Shorter  OS  *p* < 0.001 | Independent  prognostic factor  *p* = 0.011 | Tumor suppressive role: pituitary tumors, neuroblastoma, meningioma, glioma, non-small cell lung cancer, and colorectal cancer | RT-PCR, PA, AA, WB, LUCA | (Gao and Lu, 2016, 2017) |
| MIR7-3HG | 19p13.3 | Overexpressed | *miR-27a-3p/PEG10* network | RB cell lines (Weri-Rb1, Y79) and tumor tissues | Promotes proliferation  Inhibits cell apoptosis | - | - | Oncogenic role: endometrial cancer | RT-PCR, PA, AA, WB, LUCA | (Ding et al., 2020) |
| MT1JP | 16q13 | Underexpressed | *Wnt/β-catenin* pathway, *cyclin D1* and *c-Myc* | RB cell lines (Weri-Rb1, Y79) and tumor tissues | Hinders proliferation, metastasis, invasion,  and EMT | Shorter  OS  *p* = 0.002 | Independent  prognostic factor  *p* = 0.008 | Oncogenic role: hepatoblastoma, colon cancer, breast cancer, prostate cancer,  and non-small cell lung cancer | RT-PCR, PA, MA, IA, AA, WB, LUCA | (Bi et al., 2018) |
| NEAT1 | 11q13.1 | Overexpressed | *miR-204/CXCR4* axis and *miR-124* | RB cell lines (Weri-Rb1, Y79, SO-RB50) and tumor tissues | Promotes proliferation, and migration  Inhibits cell apoptosis | - | - | Oncogenic role: hepatocellular carcinoma, lung  cancer, oesophageal cancer, colorectal cancer, renal cell carcinoma,  ovarian cancer, breast cancer, and glioma | RT-PCR, PA, MA, AA, WB, LUCA, ATM | (Wang et al., 2019a; Zhong et al., 2019) |
| PANDAR | 6p21.2 | Overexpressed | *Bcl-2/caspase-3* pathway | RB cell lines (HXO-RB44, Y79, SO-RB50, Weri-Rb1) and tumor tissues | Promotes proliferation  Inhibits cell apoptosis | - | - | Oncogenic role: pancreatic ductal adenocarcinoma, hepatocellular carcinoma,  bladder cancer,  cholangiocarcinoma, and gastric cancer | RT-PCR, PA, AA, WB, LUCA, IHC, ATM | (Sheng et al., 2018) |
| PlncRNA-1 | 21q22.12 | Overexpressed | *CBR3* | RB cell lines (Weri-Rb1, Y79) and tumor tissues | Promotes proliferation, migration, and invasion | - | - | Oncogenic role:  prostate cancer, colorectal cancer, hepatocellular carcinoma, oesophageal  squamous cell carcinoma, and gastric cancer | RT-PCR, PA, MA, IA, WB | (Wang et al., 2018b) |
| PVT1 | 8q24.21 | Overexpressed | *miR-488-3p/Notch2* pathway | RB cell lines (HXO-RB44, Y79, SO-RB50, Weri-Rb1) and tumor tissues | Promotes proliferation, migration, and invasion  Inhibits cell apoptosis | Shorter  OS  *p* < 0.001 | - | Oncogenic role: renal cell carcinoma, lung cancer, cervical cancer, and breast cancer | RT-PCR, PA, MA, IA, AA, WB, LUCA, ATM | (Wu et al., 2019) |
| SNHG14 | 15q11.2 | Overexpressed | *miR-124/STAT3* pathway | RB cell lines (Weri-Rb1, Y79, SO-RB50) and tumor tissues | Promotes proliferation, migration, and invasion  Inhibits cell apoptosis | Shorter  OS  *p* = 0.032 | - | Oncogenic role: non-small cell lung cancer, cervical cancer, ovarian cancer, breast cancer, bladder cancer, and gastric cáncer  Tumor suppresive role: glioma | RT-PCR, PA, MA, IA, AA, WB, LUCA, ATM | (Sun et al., 2020) |
| SNHG16 | 17q25.1 | Overexpressed | *miR-140-5p* | RB cell lines (Weri-Rb1, Y79, SO-RB50) and tumor tissues | Promotes proliferation, and colony  formation  Inhibits cell apoptosis | - | - | Oncogenic role: glioma, pancreatic cancer, osteosarcoma, bladder cancer, non-small lung cancer, papillary  thyroid cancer, cervical cancer, gastric cancer, hepatocellular  carcinoma, and breast cancer | RT-PCR, PA, AA, LUCA, ATM | (Xu et al., 2019) |
| TCL6 | 14q32.13 | Underexpressed | *miR-21/PTEN/PI3K/Akt* pathway | RB cell lines (Weri-Rb1, Y79) and tumor tissues | Inhibits proliferation  Promotes cell apoptosis | - | - | Tumor suppressive role: clear cell renal cell carcinoma | RT-PCR, PA, AA, WB, LUCA | (Tao et al., 2019) |
| THOR | 2q14.2 | Overexpressed | *c-Myc/IGF2BP1* interaction | RB cell lines (Weri-Rb1, Y79) and tumor tissues | Promotes cell growth, migration, and  inhibits cell apoptosis in retinoblastoma Y79 cells | - | - | Oncogenic role: melanoma, non-small cell lung  cancer, osteosarcoma, and renal cell carcinoma | RT-PCR, PA, MA, AA, WB, ATM | (Shang, 2018) |
| TP73-AS1 | 1p36.32 | Overexpressed | *miR-139-3p* and *miR-874-3p/TFAP2B/ Wnt/β-catenin* pathway | RB cell lines (HXO-RB44, Y79, SO-RB50, Weri-Rb1) and tumor tissues | Promotes proliferation, metastasis, and invasion | Shorter  OS  *p* = 0.0016 | - | Oncogenic role: osteosarcoma, glioma, breast cancer | RT-PCR, PA, MA, IA, AA, WB, LUCA | (Xia et al., 2019; Wang et al., 2020a) |
| UCA1 | 19p13.12 | Overexpressed | *miR-513a-5p/STMN1* axis | RB cell lines (SO-RB50) and tumor tissues | Promotes  proliferation, and multidrug resistance | Shorter  OS  *p* = 0.0084 | - | Oncogenic role: bladder carcinoma, gastric cancer,  lung cancer, prostate cancer, breast cancer, thyroid cancer,  and oesophageal cancer | RT-PCR, PA, WB, LUCA | (Yang et al., 2020) |
| XIST | Xq13.2 | Overexpressed | *miR-101/ZEB1-ZEB2, miR-124/STAT3* and *miR-140-5p/SOX4* axis | RB cell lines (HXO-RB44, Y79, SO-RB50, Weri-Rb1) and tumor tissues | Promotes proliferation, migration, invasion, and EMT  Inhibits cell apoptosis | - | - | Oncogenic role: colorectal cancer, pancreatic cancer, non-small cell lung  cancer, osteosarcoma, hapatocelullar carcinoma, gastric cancer, and bladder cancer  Tumor suppressive role: prostate cancer, and breast cancer | RT-PCR, PA, MA, IA, AA, WB, LUCA | (Hu et al., 2018; Cheng et al., 2019; Wang et al., 2020c) |
| ZFPM2-AS1 | 8q23.1 | Overexpressed | *miR-515/HOXA1/ Wnt/β-catenin* axis | RB cell lines (Weri-Rb1, Y79, SO-RB50) and tumor tissues | Pomotes cell viability,  migration, and invasion | Shorter  OS  *p* = 0.006 | - | Oncogenic role: renal cell cancer, lung adenocarcinoma, and gastric carcinogenesis | RT-PCR, PA, MA, IA, AA, WB, LUCA, IF, IHC, ATM | (Lyv et al., 2020) |

Abbreviations: EMT: epithelial-mesenchymal transition; RT-PCR: real time polymerase chain reaction; PA: proliferation in vitro assay; MA: migration in vitro assay; DFS: disease-free survival; OS: overall survival; IA: invasion in vitro assay; AA: apoptosis in vitro assay; WB: western blot immunodetection; LUCA: luciferase expression assays; IF: immunofluorescence; IHC: immunohistochemistry; ATM: animal tumor models.**^†^** Information obtained from GeneCards database. ([www.genecards.org](about:blank)).

**REFERENCES**

Bi, L.-L., Han, F., Zhang, X.-M., and Li, Y.-Y. (2018). LncRNA MT1JP acts as a tumor inhibitor via reciprocally regulating Wnt/β-Catenin pathway in retinoblastoma. *Eur. Rev. Med. Pharmacol. Sci.* 22, 4204–4214. doi:10.26355/eurrev_201807_15414.

Cheng, Y., Chang, Q., Zheng, B., Xu, J., Li, H., and Wang, R. (2019). LncRNA XIST promotes the epithelial to mesenchymal transition of retinoblastoma via sponging miR-101. *Eur. J. Pharmacol.* 843, 210–216. doi:10.1016/j.ejphar.2018.11.028.

Ding, F., Jiang, K., Sheng, Y., Li, C., and Zhu, H. (2020). LncRNA MIR7-3HG executes a positive role in retinoblastoma progression via modulating miR-27a-3p/PEG10 axis. *Exp. Eye Res.* 193, 107960. doi:10.1016/j.exer.2020.107960.

Dong, C., Liu, S., Lv, Y., Zhang, C., Gao, H., Tan, L., et al. (2016). Long non-coding RNA HOTAIR regulates proliferation and invasion via activating Notch signalling pathway in retinoblastoma. *J. Biosci.* 41, 677–687. doi:10.1007/s12038-016-9636-7.

Gao, Y., and Lu, X. (2016). Decreased expression of MEG3 contributes to retinoblastoma progression and affects retinoblastoma cell growth by regulating the activity of Wnt/β-catenin pathway. *Tumour Biol. J. Int. Soc. Oncodevelopmental Biol. Med.* 37, 1461–1469. doi:10.1007/s13277-015-4564-y.

Gao, Y., and Lu, X. (2017). LncRNA-MEG3 mediated apoptosis of retinoblastoma by regulating P53 pathway. *Recent Adv. Ophthalmol.* 37, 301–304.

Han, N., Zuo, L., Chen, H., Zhang, C., He, P., and Yan, H. (2019). Long non-coding RNA homeobox A11 antisense RNA (HOXA11-AS) promotes retinoblastoma progression via sponging miR-506-3p. *OncoTargets Ther.* 12, 3509–3517. doi:10.2147/OTT.S195404.

Hao, F., Mou, Y., Zhang, L., Wang, S., and Yang, Y. (2018). LncRNA AFAP1-AS1 is a prognostic biomarker and serves as oncogenic role in retinoblastoma. *Biosci. Rep.* 38. doi:10.1042/BSR20180384.

Hu, C., Liu, S., Han, M., Wang, Y., and Xu, C. (2018). Knockdown of lncRNA XIST inhibits retinoblastoma progression by modulating the miR-124/STAT3 axis. *Biomed. Pharmacother. Biomedecine Pharmacother.* 107, 547–554. doi:10.1016/j.biopha.2018.08.020.

Huang, J., Yang, Y., Fang, F., and Liu, K. (2018). MALAT1 modulates the autophagy of retinoblastoma cell through miR-124-mediated stx17 regulation. *J. Cell. Biochem.* 119, 3853–3863. doi:10.1002/jcb.26464.

Li, L., Chen, W., Wang, Y., Tang, L., and Han, M. (2018a). Long non-coding RNA H19 regulates viability and metastasis, and is upregulated in retinoblastoma. *Oncol. Lett.* 15, 8424–8432. doi:10.3892/ol.2018.8385.

Li, S., Wen, D., Che, S., Cui, Z., Sun, Y., Ren, H., et al. (2018b). Knockdown of long noncoding RNA 00152 (LINC00152) inhibits human retinoblastoma progression. *OncoTargets Ther.* 11, 3215–3223. doi:10.2147/OTT.S160428.

Liu, S., Yan, G., Zhang, J., and Yu, L. (2018). Knockdown of Long Noncoding RNA (lncRNA) Metastasis-Associated Lung Adenocarcinoma Transcript 1 (MALAT1) Inhibits Proliferation, Migration, and Invasion and Promotes Apoptosis by Targeting miR-124 in Retinoblastoma. *Oncol. Res.* 26, 581–591. doi:10.3727/096504017X14953948675403.

Liu, X., Li, X., and Li, J. (2021). Long Non-coding RNA FEZF1-AS1 Promotes Growth and Reduces Apoptosis Through Regulation of miR-363-3p/PAX6 Axis in Retinoblastoma. *Biochem. Genet.* 59, 637–651. doi:10.1007/s10528-020-10026-7.

Lyv, X., Wu, F., Zhang, H., Lu, J., Wang, L., and Ma, Y. (2020). Long Noncoding RNA ZFPM2-AS1 Knockdown Restrains the Development of Retinoblastoma by Modulating the MicroRNA-515/HOXA1/Wnt/β-Catenin Axis. *Invest. Ophthalmol. Vis. Sci.* 61, 41. doi:10.1167/iovs.61.6.41.

Meng, X., Zhang, Y., Hu, Y., Zhong, J., Jiang, C., and Zhang, H. (2021). LncRNA CCAT1 sponges miR-218-5p to promote EMT, cellular migration and invasion of retinoblastoma by targeting MTF2. *Cell. Signal.* 86, 110088. doi:10.1016/j.cellsig.2021.110088.

Qi, D., Wang, M., and Yu, F. (2019). Knockdown of lncRNA-H19 inhibits cell viability, migration and invasion while promotes apoptosis via microRNA-143/RUNX2 axis in retinoblastoma. *Biomed. Pharmacother. Biomedecine Pharmacother.* 109, 798–805. doi:10.1016/j.biopha.2018.10.096.

Quan, L.-J., and Wang, W.-J. (2019). FEZF1-AS1 functions as an oncogenic lncRNA in retinoblastoma. *Biosci. Rep.* 39, BSR20190754. doi:10.1042/BSR20190754.

Shang, W., Yang, Y., Zhang, J., and Wu, Q. (2018). Long noncoding RNA BDNF-AS is a potential biomarker and regulates cancer development in human retinoblastoma. *Biochem. Biophys. Res. Commun.* 497, 1142–1148. doi:10.1016/j.bbrc.2017.01.134.

Shang, Y. (2018). LncRNA THOR acts as a retinoblastoma promoter through enhancing the combination of c-myc mRNA and IGF2BP1 protein. *Biomed. Pharmacother. Biomedecine Pharmacother.* 106, 1243–1249. doi:10.1016/j.biopha.2018.07.052.

Sheng, L., Wu, J., Gong, X., Dong, D., and Sun, X. (2018). SP1-induced upregulation of lncRNA PANDAR predicts adverse phenotypes in retinoblastoma and regulates cell growth and apoptosis in vitro and in vivo. *Gene* 668, 140–145. doi:10.1016/j.gene.2018.05.065.

Su, S., Gao, J., Wang, T., Wang, J., Li, H., and Wang, Z. (2015). Long non-coding RNA BANCR regulates growth and metastasis and is associated with poor prognosis in retinoblastoma. *Tumour Biol. J. Int. Soc. Oncodevelopmental Biol. Med.* 36, 7205–7211. doi:10.1007/s13277-015-3413-3.

Sun, X., Shen, H., Liu, S., Gao, J., and Zhang, S. (2020). Long noncoding RNA SNHG14 promotes the aggressiveness of retinoblastoma by sponging microRNA‑124 and thereby upregulating STAT3. *Int. J. Mol. Med.* 45, 1685–1696. doi:10.3892/ijmm.2020.4547.

Tao, S., Wang, W., Liu, P., Wang, H., and Chen, W. (2019). Long non-coding RNA T-cell leukemia/lymphoma 6 serves as a sponge for miR-21 modulating the cell proliferation of retinoblastoma through PTEN. *Korean J. Physiol. Pharmacol. Off. J. Korean Physiol. Soc. Korean Soc. Pharmacol.* 23, 449–458. doi:10.4196/kjpp.2019.23.6.449.

Wang, J.-X., Yang, Y., and Li, K. (2018a). Long noncoding RNA DANCR aggravates retinoblastoma through miR-34c and miR-613 by targeting MMP-9. *J. Cell. Physiol.* 233, 6986–6995. doi:10.1002/jcp.26621.

Wang, L., Wang, C., Wu, T., and Sun, F. (2020a). Long non-coding RNA TP73-AS1 promotes TFAP2B-mediated proliferation, metastasis and invasion in retinoblastoma via decoying of miRNA-874-3p. *J. Cell Commun. Signal.* 14, 193–205. doi:10.1007/s12079-020-00550-x.

Wang, L., Yang, D., Tian, R., and Zhang, H. (2019a). NEAT1 promotes retinoblastoma progression via modulating miR-124. *J. Cell. Biochem.* 120, 15585–15593. doi:10.1002/jcb.28825.

Wang, L., Zhang, Y., and Xin, X. (2020b). Long non-coding RNA MALAT1 aggravates human retinoblastoma by sponging miR-20b-5p to upregulate STAT3. *Pathol. Res. Pract.* 216, 152977. doi:10.1016/j.prp.2020.152977.

Wang, S., Liu, J., Yang, Y., Hao, F., and Zhang, L. (2018b). PlncRNA-1 is overexpressed in retinoblastoma and regulates retinoblastoma cell proliferation and motility through modulating CBR3. *IUBMB Life* 70, 969–975. doi:10.1002/iub.1886.

Wang, X., Zhang, X., Han, Y., Wang, Q., Ren, Y., Wang, B., et al. (2019b). Silence of lncRNA ANRIL represses cell growth and promotes apoptosis in retinoblastoma cells through regulating miR-99a and c-Myc. *Artif. Cells Nanomedicine Biotechnol.* 47, 2265–2273. doi:10.1080/21691401.2019.1623229.

Wang, Y., Sun, D., Sheng, Y., Guo, H., Meng, F., and Song, T. (2020c). XIST promotes cell proliferation and invasion by regulating miR-140-5p and SOX4 in retinoblastoma. *World J. Surg. Oncol.* 18, 49. doi:10.1186/s12957-020-01825-8.

Wu, X.-Z., Cui, H.-P., Lv, H.-J., and Feng, L. (2019). Knockdown of lncRNA PVT1 inhibits retinoblastoma progression by sponging miR-488-3p. *Biomed. Pharmacother. Biomedecine Pharmacother.* 112, 108627. doi:10.1016/j.biopha.2019.108627.

Xia, Z., Yang, X., Wu, S., Feng, Z., Qu, L., Chen, X., et al. (2019). LncRNA TP73-AS1 down-regulates miR-139-3p to promote retinoblastoma cell proliferation. *Biosci. Rep.* 39. doi:10.1042/BSR20190475.

Xu, C., Hu, C., Wang, Y., and Liu, S. (2019). Long noncoding RNA SNHG16 promotes human retinoblastoma progression via sponging miR-140-5p. *Biomed. Pharmacother. Biomedecine Pharmacother.* 117, 109153. doi:10.1016/j.biopha.2019.109153.

Yan, G., Su, Y., Ma, Z., Yu, L., and Chen, N. (2019). Long Noncoding RNA LINC00202 Promotes Tumor Progression by Sponging miR-3619-5p in Retinoblastoma. *Cell Struct. Funct.* 44, 51–60. doi:10.1247/csf.18033.

Yang, G., Fu, Y., Lu, X., Wang, M., Dong, H., and Li, Q. (2018). LncRNA HOTAIR/miR-613/c-met axis modulated epithelial-mesenchymal transition of retinoblastoma cells. *J. Cell. Mol. Med.* 22, 5083–5096. doi:10.1111/jcmm.13796.

Yang, L., Zhang, L., Lu, L., and Wang, Y. (2020). lncRNA UCA1 Increases Proliferation and Multidrug Resistance of Retinoblastoma Cells Through Downregulating miR-513a-5p. *DNA Cell Biol.* 39, 69–77. doi:10.1089/dna.2019.5063.

Yang, Y., and Peng, X.-W. (2018). The silencing of long non-coding RNA ANRIL suppresses invasion, and promotes apoptosis of retinoblastoma cells through the ATM-E2F1 signaling pathway. *Biosci. Rep.* 38. doi:10.1042/BSR20180558.

Zhang, A., Shang, W., Nie, Q., Li, T., and Li, S. (2018). Long non-coding RNA H19 suppresses retinoblastoma progression via counteracting miR-17-92 cluster. *J. Cell. Biochem.* 119, 3497–3509. doi:10.1002/jcb.26521.

Zhang, G., Yang, W., Li, D., Li, X., Huang, J., Huang, R., et al. (2020). lncRNA FEZF1‑AS1 promotes migration, invasion and epithelial‑mesenchymal transition of retinoblastoma cells by targeting miR‑1236‑3p. *Mol. Med. Rep.* 22, 3635–3644. doi:10.3892/mmr.2020.11478.

Zhang, H., Zhong, J., Bian, Z., Fang, X., Peng, Y., and Hu, Y. (2017). Long non-coding RNA CCAT1 promotes human retinoblastoma SO-RB50 and Y79 cells through negative regulation of miR-218-5p. *Biomed. Pharmacother. Biomedecine Pharmacother.* 87, 683–691. doi:10.1016/j.biopha.2017.01.004.

Zhao, Y., Wang, Z., Gao, M., Wang, X., Feng, H., Cui, Y., et al. (2021). lncRNA MALAT1 regulated ATAD2 to facilitate retinoblastoma progression via miR-655-3p. *Open Med. Wars. Pol.* 16, 931–943. doi:10.1515/med-2021-0290.

Zhong, W., Yang, J., Li, M., Li, L., and Li, A. (2019). Long noncoding RNA NEAT1 promotes the growth of human retinoblastoma cells via regulation of miR-204/CXCR4 axis. *J. Cell. Physiol.* 234, 11567–11576. doi:10.1002/jcp.27812.
