# Supplementary material for: Applications of Non-Coding RNAs in Patients With Retinoblastoma
Source: Front Genet. 2022 Mar 31;13:842509. doi: 10.3389/fgene.2022.842509 (PMC9008704; doi:10.3389/fgene.2022.842509)
Supplement: Supplementary file 2 [file Table2.docx]

Table 2S: miRNAs with RB tumor suppressor action.

| **miRNA** | **Location^†^** | **Expression** | **Target Mechanism** | **Sample Type** | **Suppressive Events** | **Survival** | **Prognostic Factor** | **Role** **in Other Cancers** | **Validation Method** | **Reference** |
| --- | --- | --- | --- | --- | --- | --- | --- | --- | --- | --- |
| mir-29a | 7q32.3 | Underexpressed | *STAT3* | RB cell lines (SO-RB50, Y79) and tumor tissues | Cell proliferation, migration, and invasion  Induces cell apoptosis | - | - | Oncogenic role: breast cancer, colorectal cancer, and gastric cancer  Tumor suppressive role: non‑small cell  lung cancer, hepatocellular carcinoma, and papillary thyroid carcinoma | RT-PCR, PA, MA, IA, AA, WB, LUCA, ATM | (Liu et al., 2018) |
| miR-31 | 9p21.3 | Underexpressed | *STK40, PPP6C* and *DLL3* | RB cell lines (Weri-Rb1, Y79) and tumor tissues | Cell proliferation (retinoblastoma Y79 cells)  Induces cell apoptosis (retinoblastoma Y79 cells) | - | - | Tumor suppressive role: ovary cancer, pancreas cancer, and brain cancers | RT-PCR, PA, AA, WB, LUCA, IF, IHC | (Montoya et al., 2015) |
| miR-34a | 1p36.22 | Underexpressed | *HMGB1*, *Notch1* and *MAGE-A/p53 axis* | RB cell lines (Weri-Rb1, Y79, SO-RB50, HXO-RB44) and tumor tissues | Cell viability, proliferation, autophagy, and EMT  Induces cell apoptosis | Longer  OS  *p* < 0.05 | - | Tumor suppressive role: glioblastoma multiforme | RT-PCR, PA, AA, WB, LUCA | (Liu et al., 2014; Yang et al., 2019; Yin et al., 2020) |
| miR-98 | Xp11.22 | Underexpressed | *IGF1R/k-Ras/Raf/MEK/ERK* pathway | RB cell lines (Weri-Rb1, Y79, SO-RB50) and tumor tissues | Cell proliferation, migration, and invasion  Induces cell apoptosis | Longer  OS  *p* = 0.0131 | - | Tumor suppressive role:  prostate cancer, head and neck squamous cell carcinoma, and breast cancer | RT-PCR, PA, MA, IA, AA, WB, LUCA, IHC | (Guo et al., 2019) |
| miR-101 | 1p31.3 | Underexpressed | *EZH2* | RB cell lines (Weri-Rb1, Y79) and tumor tissues | Cell proliferation  Induces cell apoptosis | - | - | Tumor suppressive role: gastric cancer, endometrial serous adenocarcinoma, human hepatocellular carcinoma, and prostate cancer | RT-PCR, PA, AA, LUCA | (Lei et al., 2014) |
| miR-125a-5p | 19q13.41 | Underexpressed | *TAZ-EGFR* pathway | RB cell lines (Weri-Rb1, Y79) and tumor tissues | Cell proliferation | - | Suggested by data:  cTNM stage (*p* = 0.015) and  cell differentiation (*p* = 0.022) | Tumor suppressive role: glioblastoma, lung cancer, and breast cancer | RT-PCR, PA, WB, LUCA, IHC, ATM | (Zhang et al., 2016) |
| miR-138-5p | 3p21.32 | Underexpressed | *PDK1*, *SLC7A5* and *PEG10* | RB cell lines (Weri-Rb1, Y79, SO-RB50, HXO-RB44) and tumor tissues | Cell proliferation, migration, and invasion  Induces cell apoptosis | - | - | Tumor suppressive role: bladder cancer, non-small cell lung cancer, and pancreatic carcinoma | RT-PCR, PA, MA, IA, AA, WB, LUCA, IHC, ATM | (Wang et al., 2017; Zhang et al., 2021; Zheng et al., 2021) |
| miR-183 | 7q32.2 | Underexpressed | *LRP6* | RB cell lines (Weri-Rb1, Y79, SO-RB50) and tumor tissues | Cell proliferation, migration, and invasion  Induces cell apoptosis | - | - | Oncogenic role:  colorectal cancer,  melanoma, and prostate cancer  Tumor suppressive role:  hepatocellular carcinoma,  osteosarcoma, lung cancer, and breast cancer | RT-PCR, PA, MA, IA, AA, WB, LUCA, IF, ATM | (Wang et al., 2014) |
| miR-184 | 15q25.1 | Underexpressed | *SLC7A5/ATR/ATM* pathway | RB cell lines (Weri-Rb1, Y79) and tumor tissues | Cell proliferation, migration, and invasion  Induces cell apoptosis | - | - | Tumor suppressive role:  malignant glioma, and breast cancer | RT-PCR, PA, MA, IA, AA, WB, LUCA, IF, IHC | (He et al., 2019) |
| miR-186 | 1p31.1 | Underexpressed | *DIXDC1* | RB cell lines (Weri-Rb1, Y79, SO-RB50, HXO-RB44) and tumor tissues | Cell proliferation, and invasion | - | - | Tumor suppressive role:  lung cancer, epithelial ovarian cancer, bladder cancer, multiple mieloma, myeloid leukemia, oesophageal squamous cell carcinoma, gastric cancer, prostate cancer, and colon cancer | RT-PCR, PA, IA, WB, LUCA | (Che et al., 2018) |
| miR-200a | 1p36.33 | Underexpressed | *ACOT7* and *DLL3* | RB cell lines (Weri-Rb1, Y79) and tumor tissues | Cell proliferation (retinoblastoma Y79 cells)  Induces cell apoptosis (retinoblastoma Y79 cells) | - | - | Tumor suppressive role: meningioma | RT-PCR, PA, AA, WB, LUCA, IF, IHC | (Montoya et al., 2015) |
| miR-204 | 9q21.12 | Underexpressed | *Cyclin D2* and *MMP-9* | RB cell lines (Weri-Rb1, Y79, SO-RB50) and tumor tissues | Cell proliferation, migration, and invasion | - | - | - | RT-PCR, PA, MA, IA, WB, LUCA, ATM | (Wu et al., 2015) |
| miR-214-3p | 1q24.3 | Underexpressed | *ABCB1* and *XIAP* | RB cell lines (Weri-Rb1, Y79, SO-RB50) and tumor tissues | Cell proliferation  Induces cell apoptosis | Longer  OS  *p* = 0.0044 | - | Oncogenic role: osteosarcoma, and bladder cancer  Tumor suppressive role:  oral squamous cell, oesophageal  squamous cancer, endometrial carcinoma, breast cancer, lung cancer, and hepatocellular  carcinoma | RT-PCR, PA, AA, WB, LUCA, ATM | (Yang et al., 2020) |
| miR-218-5p | 4p15.31 | Underexpressed | *NACC1/Akt/mTOR* axis and *MTF2* | RB cell lines (Weri-Rb1, Y79, SO-RB50) and tumor tissues | Cell proliferation, migration, and invasion  Induces cell apoptosis | - | - | Tumor suppressive role:  lung cancer, breast cancer, liver cancer, gallbladder cancer, colorectal  cancer, prostate cancer, and gastric cancer | RT-PCR, PA, MA, IA, AA, WB, LUCA | (Li et al., 2020; Meng et al., 2021) |
| miR-330 | 19q13.32 | Underexpressed | *ROCK1* | RB cell lines (Weri-Rb1, Y79, SO-RB50) and tumor tissues | Cell proliferation, and invasion | - | - | Oncogenic role: breast cancer, oesophageal cancer, non‑small cell lung  Cancer, and glioblastoma  Tumor suppressive role: prostate cancer,  Osteosarcoma, and colorectal cancer | RT-PCR, PA, IA, WB, LUCA | (Wang et al., 2019b) |
| miR-361-3p | Xq21.2 | Underexpressed | *GLI1, GLI3* and *SHH* pathway | RB cell lines (Weri-Rb1, Y79), tumor tissues and serum samples | Cell proliferation | - | - | Oncogenic role: cervical cancer  Tumor suppressive role: breast cancer, non‑small cell lung cancer, cutaneous squamous cell carcinoma, prostate cancer, colorectal cancer, gastric cancer, and hepatocellular carcinoma | RT-PCR, PA, WB, LUCA | (Zhao and Cui, 2019) |
| miR-365b-3p | 17q11.2 | Underexpressed | *PAX6* | RB cell lines (Weri-Rb1, Y79, SO-RB50) and tumor tissues | Cell proliferation  Induces cell apoptosis | - | - | Tumor suppressive role: colon cancer | RT-PCR, PA, AA, WB, IF, ATM | (Wang et al., 2013) |
| miR-485 | 14q32.31 | Underexpressed | *Wnt3a* and *Wnt/β-catenin* pathway | RB cell lines (Weri-Rb1, Y79, SO-RB50) and tumor tissues | Cell proliferation, migration, and invasion  Induces cell apoptosis | - | - | Tumor suppressive role: colorectal cancer, gastric cancer, glioblastoma, hepatocellular carcinoma,  lung adenocarcinoma, breast cancer, melanoma, oral  tongue squamous cell carcinoma, and bladder  cancer | RT-PCR, PA, MA, IA, AA, WB, LUCA, ATM | (Lyu et al., 2019) |
| miR-504 | Xq26.3 | Underexpressed | *AEG-1* | RB cell lines (Weri-Rb1, Y79, SO-RB50) and tumor tissues | Cell proliferation, and invasion | - | - | Oncogenic role: nasopharyngeal carcinoma  Tumor suppressive role: glioma, hypopharyngeal squamous cell carcinoma, and oral squamous cell carcinoma | RT-PCR, PA, IA, WB, LUCA | (Wang et al., 2019a) |

Abbreviations: RT-PCR: real time polymerase chain reaction; PA: proliferation in vitro assay; MA: migration in vitro assay; OS: overall survival; IA: invasion in vitro assay; AA: apoptosis in vitro assay; WB: western blot immunodetection; LUCA: luciferase expression assays; IF: immunofluorescence; IHC: immunohistochemistry; ATM: animal tumor models.**^†^** Information obtained from GeneCards database ([www.genecards.org](about:blank)).

Table 3S: miRNAs with RB carcinogenic action.

| **miRNA** | **Location^†^** | **Expression** | **Target Mechanism** | **Sample Type** | **Oncogenic Events** | **Survival** | **Prognostic Factor** | **Role** **in Other Cancers** | **Validation Method** | **Reference** |
| --- | --- | --- | --- | --- | --- | --- | --- | --- | --- | --- |
| miR-17-92 cluster | 13q31.3 | Overexpressed | *EpCAM* and *STAT3* | RB cell lines (Y79) and tumor tissues | Cell proliferation, and invasion  Represses cell apoptosis | - | - | Oncogenic role: hepatocellular cancer | RT-PCR, PA, IA, AA, WB, IHC, ATM | (Kandalam et al., 2012; Jo et al., 2014) |
| miR-21 | 17q23.1 | Overexpressed | *PDCD4*, *Bax*, *Bcl-2* and *PTEN/PI3K/Akt* pathway | RB cell lines (Weri-Rb1, Y79, Rb355) and tumor tissues | Cell viability, proliferation, migration, and invasion  Represses cell apoptosis | - | - | Oncogenic role:  breast cancer, glioblastoma, glioma, colorectal cancer, prostate cancer,  hepatocellular cancer, non-small cell lung cancer, papillary thyroid carcinoma, gastric cancer, osteosarcoma, diffuse large B-cell lymphoma, and laryngeal carcinoma | RT-PCR, PA, MA, IA, AA, WB | (Shen et al., 2014; Gui et al., 2016) |
| miR-25-3p | 7q22.1 | Overexpressed | *PTEN/Akt* pathway | RB cell lines (Weri-Rb1, Y79, SO-RB50) and tumor tissues | Cell proliferation, migration, and invasion  Represses cell apoptosis | - | - | Oncogenic role:  renal tumors, triple negative  breast cancer, melanoma, and non-small  cell lung cancer | RT.PCR, PA, MA, IA, AA, WB, LUCA, IF, IHC, ATM | (Wan et al., 2019) |
| miR-106b | 7q22.1 | Overexpressed | *RUNX3* and *ZBTB4* | RB cell lines (Weri-Rb1, Y79) and tumor tissues | Cell proliferation, migration, and invasion  Represses cell apoptosis | - | - | - | RT-PCR, PA, MA, IA, AA, WB, LUCA | (Yang et al., 2017; Bu et al., 2018) |
| miR-125b | 11q24.1 | Overexpressed | *DRAM2* | RB cell lines (HXO-RB44, Y79, SO-RB50) and tumor tissues | Cell proliferation, migration, and invasion  Represses cell apoptosis | - | - | Oncogenic role: prostate cancer,  non-small-cell lung cancer, and oral squamous cell carcinoma  . | RT-PCR, PA, MA, IA, AA, WB, LUCA | (Bai et al., 2016) |
| miR-130b | 22q11.21 | Overexpressed | *EpCAM* | RB cell lines (Weri-Rb1, Y79) and tumor tissues | Cell proliferation, and invasion  Represses cell apoptosis | - | - | Oncogenic role: colorectal cancer, gastric cancer, and renal carcinoma | RT-PCR, PA, IA, AA, WB | (Beta et al., 2014) |
| miR-181c | 19p13.12 | Overexpressed | *EpCAM* | RB cell lines (Weri-Rb1, Y79) and tumor tissues | Cell proliferation, and invasion  Represses cell apoptosis | - | - | Oncogenic role: hepatocellular  carcinoma | RT-PCR, PA, IA, AA, WB | (Beta et al., 2014) |
| miR-224-3p | Xq28 | Overexpressed | *LATS2/Hippo-YAP* axis | RB cell lines (Y79) and tumor tissues | Cell proliferation, and angiogenesis  Represses cell apoptosis | - | - | Oncogenic role: high-risk human papillomavirus–positive cervical cancer | RT-PCR, PA, AA, WB, LUCA, IHC, ATM | (Song et al., 2020) |
| miR-492 | 12q22 | Overexpressed | *LATS2* | RB cell lines (Weri-Rb1, Y79, SO-RB50) and tumor tissues | Cell proliferation, and invasion | - | - | Oncogenic role: hepatoblastoma, breast cancer, and hepatic cancer  Tumor suppressive role: cervical cancer, osteosarcoma, and clear cell renal cell carcinoma | RT-PCR, PA, IA, WB, LUCA | (Sun et al., 2019) |
| miR-494 | 14q32.31 | Overexpressed | *PTEN/PI3K/Akt* pathway | RB cell lines (SO-RB50, Y79) and tumor tissues | Cell proliferation, migration, and invasion | - | - | Oncogenic role: lung cancer, endometrial cancer, nasopharyngeal carcinoma, gastric cancer,  cervical cancer, and colorectal cancer | RT-PCR, PA, MA, IA, WB, LUCA | (Xu et al., 2020) |

Abbreviations: RT-PCR: real time polymerase chain reaction; PA: proliferation in vitro assay; MA: migration in vitro assay; OS: overall survival; IA: invasion in vitro assay; AA: apoptosis in vitro assay; WB: western blot immunodetection; LUCA: luciferase expression assays; IF: immunofluorescence; IHC: immunohistochemistry; ATM: animal tumor models. **^†^** Information obtained from GeneCards database ([www.genecards.org](about:blank)).

**REFERENCES**

Bai, S., Tian, B., Li, A., Yao, Q., Zhang, G., and Li, F. (2016). MicroRNA-125b promotes tumor growth and suppresses apoptosis by targeting DRAM2 in retinoblastoma. *Eye Lond. Engl.* 30, 1630–1638. doi:10.1038/eye.2016.189.

Beta, M., Khetan, V., Chatterjee, N., Suganeswari, G., Rishi, P., Biswas, J., et al. (2014). EpCAM knockdown alters microRNA expression in retinoblastoma--functional implication of EpCAM regulated miRNA in tumor progression. *PloS One* 9, e114800. doi:10.1371/journal.pone.0114800.

Bu, W., Wang, Y., and Min, X. (2018). MicroRNA-106b promotes the proliferation, migration and invasion of retinoblastoma cells by inhibiting the expression of ZBTB4 protein. *Exp. Ther. Med.* 16, 4537–4545. doi:10.3892/etm.2018.6811.

Che, X., Qian, Y., and Li, D. (2018). Suppression of Disheveled-Axin Domain Containing 1 (DIXDC1) by MicroRNA-186 Inhibits the Proliferation and Invasion of Retinoblastoma Cells. *J. Mol. Neurosci. MN* 64, 252–261. doi:10.1007/s12031-017-1017-7.

Gui, F., Hong, Z., You, Z., Wu, H., and Zhang, Y. (2016). MiR-21 inhibitor suppressed the progression of retinoblastoma via the modulation of PTEN/PI3K/AKT pathway. *Cell Biol. Int.* 40, 1294–1302. doi:10.1002/cbin.10678.

Guo, L., Bai, Y., Ji, S., and Ma, H. (2019). MicroRNA‑98 suppresses cell growth and invasion of retinoblastoma via targeting the IGF1R/k‑Ras/Raf/MEK/ERK signaling pathway. *Int. J. Oncol.* 54, 807–820. doi:10.3892/ijo.2019.4689.

He, T.-G., Xiao, Z.-Y., Xing, Y.-Q., Yang, H.-J., Qiu, H., and Chen, J.-B. (2019). Tumor Suppressor miR-184 Enhances Chemosensitivity by Directly Inhibiting SLC7A5 in Retinoblastoma. *Front. Oncol.* 9, 1163. doi:10.3389/fonc.2019.01163.

Jo, D. H., Kim, J. H., Cho, C. S., Cho, Y.-L., Jun, H. O., Yu, Y. S., et al. (2014). STAT3 inhibition suppresses proliferation of retinoblastoma through down-regulation of positive feedback loop of STAT3/miR-17-92 clusters. *Oncotarget* 5, 11513–11525. doi:10.18632/oncotarget.2546.

Kandalam, M. M., Beta, M., Maheswari, U. K., Swaminathan, S., and Krishnakumar, S. (2012). Oncogenic microRNA 17-92 cluster is regulated by epithelial cell adhesion molecule and could be a potential therapeutic target in retinoblastoma. *Mol. Vis.* 18, 2279–2287.

Lei, Q., Shen, F., Wu, J., Zhang, W., Wang, J., and Zhang, L. (2014). MiR-101, downregulated in retinoblastoma, functions as a tumor suppressor in human retinoblastoma cells by targeting EZH2. *Oncol. Rep.* 32, 261–269. doi:10.3892/or.2014.3167.

Li, L., Yu, H., and Ren, Q. (2020). MiR-218-5p Suppresses the Progression of Retinoblastoma Through Targeting NACC1 and Inhibiting the AKT/mTOR Signaling Pathway. *Cancer Manag. Res.* 12, 6959–6967. doi:10.2147/CMAR.S246142.

Liu, K., Huang, J., Xie, M., Yu, Y., Zhu, S., Kang, R., et al. (2014). MIR34A regulates autophagy and apoptosis by targeting HMGB1 in the retinoblastoma cell. *Autophagy* 10, 442–452. doi:10.4161/auto.27418.

Liu, S., Zhang, X., Hu, C., Wang, Y., and Xu, C. (2018). miR-29a inhibits human retinoblastoma progression by targeting STAT3. *Oncol. Rep.* 39, 739–746. doi:10.3892/or.2017.6144.

Lyu, X., Wang, L., Lu, J., Zhang, H., and Wang, L. (2019). microRNA‑485 inhibits the malignant behaviors of retinoblastoma by directly targeting Wnt3a. *Oncol. Rep.* 41, 3137–3147. doi:10.3892/or.2019.7061.

Meng, X., Zhang, Y., Hu, Y., Zhong, J., Jiang, C., and Zhang, H. (2021). LncRNA CCAT1 sponges miR-218-5p to promote EMT, cellular migration and invasion of retinoblastoma by targeting MTF2. *Cell. Signal.* 86, 110088. doi:10.1016/j.cellsig.2021.110088.

Montoya, V., Fan, H., Bryar, P. J., Weinstein, J. L., Mets, M. B., Feng, G., et al. (2015). Novel miRNA-31 and miRNA-200a-Mediated Regulation of Retinoblastoma Proliferation. *PloS One* 10, e0138366. doi:10.1371/journal.pone.0138366.

Shen, F., Mo, M.-H., Chen, L., An, S., Tan, X., Fu, Y., et al. (2014). MicroRNA-21 Down-regulates Rb1 Expression by Targeting PDCD4 in Retinoblastoma. *J. Cancer* 5, 804–812. doi:10.7150/jca.10456.

Song, L., Huang, Y., Zhang, X., Han, S., Hou, M., and Li, H. (2020). Downregulation of microRNA-224-3p Hampers Retinoblastoma Progression via Activation of the Hippo-YAP Signaling Pathway by Increasing LATS2. *Invest. Ophthalmol. Vis. Sci.* 61, 32. doi:10.1167/iovs.61.3.32.

Sun, Z., Zhang, A., and Zhang, L. (2019). Inhibition of microRNA‑492 attenuates cell proliferation and invasion in retinoblastoma via directly targeting LATS2. *Mol. Med. Rep.* 19, 1965–1971. doi:10.3892/mmr.2018.9784.

Wan, W., Wan, W., Long, Y., Li, Q., Jin, X., Wan, G., et al. (2019). MiR-25-3p promotes malignant phenotypes of retinoblastoma by regulating PTEN/Akt pathway. *Biomed. Pharmacother. Biomedecine Pharmacother.* 118, 109111. doi:10.1016/j.biopha.2019.109111.

Wang, J., Wang, X., Li, Z., Liu, H., and Teng, Y. (2014). MicroRNA-183 suppresses retinoblastoma cell growth, invasion and migration by targeting LRP6. *FEBS J.* 281, 1355–1365. doi:10.1111/febs.12659.

Wang, J., Wang, X., Wu, G., Hou, D., and Hu, Q. (2013). MiR-365b-3p, down-regulated in retinoblastoma, regulates cell cycle progression and apoptosis of human retinoblastoma cells by targeting PAX6. *FEBS Lett.* 587, 1779–1786. doi:10.1016/j.febslet.2013.04.029.

Wang, L., Lyu, X., Ma, Y., Wu, F., and Wang, L. (2019a). MicroRNA‑504 targets AEG‑1 and inhibits cell proliferation and invasion in retinoblastoma. *Mol. Med. Rep.* 19, 2935–2942. doi:10.3892/mmr.2019.9923.

Wang, L., Wang, L., Li, L., Zhang, H., and Lyu, X. (2019b). MicroRNA‑330 is downregulated in retinoblastoma and suppresses cell viability and invasion by directly targeting ROCK1. *Mol. Med. Rep.* 20, 3440–3447. doi:10.3892/mmr.2019.10545.

Wang, Z., Yao, Y.-J., Zheng, F., Guan, Z., Zhang, L., Dong, N., et al. (2017). Mir-138-5p acts as a tumor suppressor by targeting pyruvate dehydrogenase kinase 1 in human retinoblastoma. *Eur. Rev. Med. Pharmacol. Sci.* 21, 5624–5629. doi:10.26355/eurrev_201712_14005.

Wu, X., Zeng, Y., Wu, S., Zhong, J., Wang, Y., and Xu, J. (2015). MiR-204, down-regulated in retinoblastoma, regulates proliferation and invasion of human retinoblastoma cells by targeting CyclinD2 and MMP-9. *FEBS Lett.* 589, 645–650. doi:10.1016/j.febslet.2015.01.030.

Xu, F., Liu, G., Wang, L., Wang, X., Jin, X., and Bo, W. (2020). miR-494 promotes progression of retinoblastoma via PTEN through PI3K/AKT signaling pathway. *Oncol. Lett.* 20, 1952–1960. doi:10.3892/ol.2020.11749.

Yang, G., Fu, Y., Lu, X., Wang, M., Dong, H., and Li, Q. (2019). miR‑34a regulates the chemosensitivity of retinoblastoma cells via modulation of MAGE‑A/p53 signaling. *Int. J. Oncol.* 54, 177–187. doi:10.3892/ijo.2018.4613.

Yang, G., Fu, Y., Zhang, L., Lu, X., and Li, Q. (2017). miR106b regulates retinoblastoma Y79 cells through Runx3. *Oncol. Rep.* 38, 3039–3043. doi:10.3892/or.2017.5931.

Yang, L., Zhang, L., Lu, L., and Wang, Y. (2020). miR-214-3p Regulates Multi-Drug Resistance and Apoptosis in Retinoblastoma Cells by Targeting ABCB1 and XIAP. *OncoTargets Ther.* 13, 803–811. doi:10.2147/OTT.S235862.

Yin, W., Gao, F., and Zhang, S. (2020). MicroRNA‑34a inhibits the proliferation and promotes the chemosensitivity of retinoblastoma cells by downregulating Notch1 expression. *Mol. Med. Rep.* 22, 1613–1620. doi:10.3892/mmr.2020.11238.

Zhang, Y., Dou, X., Kong, Q., Li, Y., and Zhou, X. (2021). Circ_0075804 promotes the malignant behaviors of retinoblastoma cells by binding to miR-138-5p to induce PEG10 expression. *Int. Ophthalmol.* doi:10.1007/s10792-021-02067-7.

Zhang, Y., Xue, C., Zhu, X., Zhu, X., Xian, H., and Huang, Z. (2016). Suppression of microRNA-125a-5p upregulates the TAZ-EGFR signaling pathway and promotes retinoblastoma proliferation. *Cell. Signal.* 28, 850–860. doi:10.1016/j.cellsig.2016.04.002.

Zhao, D., and Cui, Z. (2019). MicroRNA-361-3p regulates retinoblastoma cell proliferation and stemness by targeting hedgehog signaling. *Exp. Ther. Med.* 17, 1154–1162. doi:10.3892/etm.2018.7062.

Zheng, T., Chen, W., Wang, X., Cai, W., Wu, F., and Lin, C. (2021). Circular RNA circ-FAM158A promotes retinoblastoma progression by regulating miR-138-5p/SLC7A5 axis. *Exp. Eye Res.* 211, 108650. doi:10.1016/j.exer.2021.108650.
