# Supplementary material for: Applications of Non-Coding RNAs in Patients With Retinoblastoma
Source: Front Genet. 2022 Mar 31;13:842509. doi: 10.3389/fgene.2022.842509 (PMC9008704; doi:10.3389/fgene.2022.842509)
Supplement: Supplementary file 3 [file Table3.DOCX]

**Contribution to the Field**

The most common primary intraocular malignancy in childhood is retinoblastoma (RB), a retinal tumor with an incidence of one case per 15,000–20,000 live births. In patients with RB, the conservation of the eyeball and survival depend largely on the tumor stage at the time of diagnosis, and RB is diagnosed substantially later in underdeveloped nations than in developed nations and results in a markedly lower survival rate. Although numerous treatment methods are available for local tumor management in RB, metastatic illness remains the most serious consequence in the patients, and demand exists for designing new therapeutic strategies. In RB development, a major role is played by the loss of the tumor-suppressor gene *RB1*, but recent studies have also revealed the involvement of epigenetic regulation in RB, particularly through mechanisms mediated by non-coding RNAs (ncRNAs). Several ncRNAs are now considered potential diagnostic biomarkers, prognostic factors, and therapeutic targets in RB, and we provide here a clinical overview of our current understanding of ncRNAs in relation to RB. Specifically, we address the functions of distinct ncRNAs reported to affect RB development, and we suggest that additional studies are necessary to comprehensively elucidate ncRNA roles in both RB pathophysiology and clinical treatment.
